# Plasmon-Enhanced Photoresponse of Self-Powered Si Nanoholes Photodetector by Metal Nanowires

**DOI:** 10.3390/nano11092460

**Published:** 2021-09-21

**Authors:** Pericle Varasteanu, Antonio Radoi, Oana Tutunaru, Anton Ficai, Razvan Pascu, Mihaela Kusko, Iuliana Mihalache

**Affiliations:** 1 National Institute for Research and Development in Microtechnologies-IMT Bucharest, 126A Erou Iancu Nicolae Street, 077190 Bucharest, Romania; pericle.varasteanu@imt.ro (P.V.); antonio.radoi@imt.ro (A.R.); oana.tutunaru@imt.ro (O.T.); 2 Faculty of Applied Chemistry and Materials Science, University Politehnica of Bucharest, Gh. Polizu St 1-7, 011061 Bucharest, Romania; anton.ficai@upb.ro; 3 Faculty of Electronics, Telecommunications and Information Technology, University Politehnica of Bucharest, 060042 Bucharest, Romania; 4 Romanian Young Academy, Research Institute of the University of Bucharest, University of Bucharest, 030018 Bucharest, Romania

**Keywords:** metal nanowires, nanostructured silicon, silicon nanoholes, plasmonic photodetector

## Abstract

In this work, we report the development of self-powered photodetectors that integrate silicon nanoholes (SiNHs) and four different types of metal nanowires (AgNWs, AuNWs, NiNWs, PtNWs) applied on the SiNHs’ surface using the solution processing method. The effectiveness of the proposed architectures is evidenced through extensive experimental and simulation analysis. The AgNWs/SiNHs device showed the highest photo-to-dark current ratio of 2.1 × 10^−4^, responsivity of 30 mA/W and detectivity of 2 × 10^11^ Jones along with the lowest noise equivalent power (NEP) parameter of 2.4 × 10^−12^ WHz^−1/2^ in the blue light region. Compared to the bare SiNHs device, the AuNWs/SiNHs device had significantly enhanced responsivity up to 15 mA/W, especially in the red and near-infrared spectral region. Intensity-modulated photovoltage spectroscopy (IMVS) measurements revealed that the AgNWs/SiNHs device generated the longest charge carrier lifetime at 470 nm, whereas the AuNWs/SiNHs showed the slowest recombination rate at 627 nm. Furthermore, numerical simulation confirmed the local field enhancement effects at the MeNWs and SiNHs interface. The study demonstrates a cost-efficient and scalable strategy to combine the superior light harvesting properties of SiNHs with the plasmonic absorption of metallic nanowires (MeNWs) towards enhanced sensitivity and spectral-selective photodetection induced by the local surface plasmon resonance effects.

## 1. Introduction

Optoelectronic devices rely heavily on silicon (Si)-based materials owing to their high performance, low cost, earth abundance and convenient compatibility with complementary metal-oxide semiconductor (CMOS) technologies [1]. Recently, the surface topography of silicon wafers patterned with nano and microstructures of various shapes such as wires, needles, cones or pyramids demonstrated high potential for photodetector [2], photovoltaic [3,4,5] and sensor [6] applications. It is well known that the patterning process is the key factor leading to interesting optical properties and electrical characteristics [7,8]. In this view, the black and textured surface of silicon is considered a superior alternative to crystalline silicon because it is able to enhance the optical absorption in the visible and near infrared region. Due to the surface nanostructures, nearly 80% of incident light is trapped and subjected to multiple reflections and transmissions, boosting the photons’ absorption ability [9,10].

The exceptional properties of structured silicon provide a powerful support for devices such as photodetectors or solar cells, especially if wide response range and high responsivity performance is targeted [11,12,13]. Currently, the research has focused on improving the spectral response range and selectivity in silicon-based devices by developing many types of heterojunctions that integrate materials from organic to narrow band-gap semiconductors, metallic thin films and nanostructures.

Surface plasmons (SPs) are generated at the metal/dielectric interface through coherent and collective oscillation of free electrons exposed to the electromagnetic field excitation. In particular, in the case of Schottky contact the excitation of surface plasmonic modes at the surface of metal provides a strong electromagnetic field that further generates hot electrons, which will migrate into the semiconductor, increasing photo-induced carriers. Therefore, the local field enhancement effect of SPs is a great way to locally concentrate light for many device applications including energy conversion and surface-enhanced Raman scattering (SERS) applications. At present, the topic of hot charge carriers’ efficient generation and extraction is of great interest and recent efforts have been made to elucidate the physical mechanism underlying charge transfer dynamics [14,15,16]. Recent studies proposed the incorporation of various metallic structures such as thin films, gratings, nanoparticles, nanorods and nanowires nearby active layers to support SP generation under random polarized light and conveniently stimulate strong absorption of light [17,18]. In this direction, hot electron photodetectors using Au nanoparticles (NPs) [19], Ag NPs [20] and Au thin film [21]-decorated silicon pyramids have demonstrated enhanced near-infrared (NIR) photoresponse and signal-to-noise ratio through the hot electron tunneling mechanism. Metallic nanostructures such as Au nanoislands directly grown on the semiconductor surface have shown femtosecond range ultrafast hot electron injection into the coupled media, as suggested by transient dynamics analysis [22]. Moreover, SPs’ effects of tuning the absorption range facilitate the design of a controllable photoresponse for the target wavelength range in optoelectronic devices [23,24,25].

Among various metallic structures, nanowires (e.g., AgNWs, AuNWs, CuNWs etc.) were initially employed as flexible transparent conductive electrodes in many display technologies, touch screens, sensors, organic light-emitting diodes (OLEDs) and solar cells owing to their low sheet resistance (RSH), reaching down to 2.5 Ω/sq for more than 85.5% optical transmittance in the visible SWIR domain (300–2000 nm) [26], mechanical characteristics, well-defined geometries and low-cost fabrication, which make them a good choice for the substitution of brittle indium tin oxide (ITO) films [27,28,29,30]. However, metallic nanowires (MeNWs) with excellent plasmonic properties hold a great potential for applications that combine the photonic detection achieved in configurations based on separation of excitons and extraction of charge carries with surface plasmon effects toward increasing photoresponse sensitivity and signal-to-noise ratio. Nevertheless, the NWs’ performance might rely on many factors such as purity, surface chemistry, deposition method and post-treatment welding processes, which must be well regarded [31,32,33,34,35,36].

Herein, we report the plasmon-enhanced photoresponse of a novel photodetector architecture based on silicon nanoholes (SiNHs) following the incorporation of four different types of metallic nanowires (AgNWs, AuNWs, NiNWs, PtNWs) onto the silicon surface. The plasmonic Schottky optical detectors show an enhanced photocurrent, responsivity and external quantum efficiency (EQE) under zero bias conditions, attributed mainly to the increased photo-induced carrier injection and high electric field distribution surrounding the nanowires, as demonstrated by the simulation results.

## 2. Materials and Methods

### 2.1. Fabrication of the Photodetector (MeNWs/SiNHs)

The reagents were purchased from Sigma-Aldrich (Darmstadt, Germany) and used without further purification. Commercially available *p*-type Si <100> wafers (SIEGERT WAFER GmbH, Aachen, Germany) with boron doping of 2 × 10^16^/cm^3^ were sonicated in ethanol, acetone and (5% *v*/*v*) Extran^®^ solution for 15 min and ultimately cleaned in 3:1 *v*/*v* H_2_SO_4_ and H_2_O_2_ solution for 30 min at room temperature. An insulator stack consisting of SiO_2_ (500 nm)/Si_3_N_4_ (176 nm) was deposited on top of Si substrate by low pressure chemical vapor deposition (LPCVD) technique. In order to achieve the bottom electrode Au/Cr bi-layer, film was deposited on the backside of the wafers by using the e-beam evaporation technique. Square windows (0.5 × 0.5 cm) were opened in the insulator layer using photolithography and reactive ion etching in order to define the active area where silicon nanoholes (SiNHs) are fabricated.

The localized SiNHs’ fabrication was based on a metal-assisted chemical etching (MACE) technique [3]. In the first step, substrates were immersed for 1 min in 4.8 M HF and 0.02 M AgNO_3_ aqueous solution at room temperature (RT). In order to remove the extra Ag+ ions, the substrates were rinsed thoroughly with DI water. In the second step, the as-prepared samples were etched in dark conditions for 5 min in a mixture of 4.8 M HF and 0.1 M H_2_O_2_, then washed with DI water. Following SiNHs fabrication, the wafers were immersed in concentrated HNO_3_ (65%) for 30 min to remove any residual catalyst.

A metallic stack consisting of Cr (10 nm)/Au (100 nm) was deposited on the frontside of the wafers as a frame defined by photolithography to obtain the contact pads. The pads were not only framing the flat Si substrate but also contacting the active area of silicon nanoholes. Finally, devices were diced into 1 × 1 cm chips and packaged using wire bonding technology in dual in-line (DIL) capsules to evaluate their opto-electrical performance.

Metal nanowires (MeNWs) fabricated using electrodeposition on an alumina template were purchased from DropSens (Metrohm DropSens S.L., Asturias, Spain). Purified metallic NWs stored in ethanol were sonicated for 30 min before deposition in order to prevent or to reduce agglomeration of nanowires. The SiNHs’ surface was treated with 2% HF solution prior to the spin-coating deposition of MeNWs. The spinning conditions were optimized to cover the SiNHs surface with uniformly distributed MeNW films.

### 2.2. Characterization and Measurements

Surface morphology was investigated using a field emission scanning electron microscope (FESEM), the FEI-Nova NanoSEM 630 (FEI Company, Hillsboro, OR, USA) equipped with EDX analyzer. Absorption spectra were investigated with a Hitachi U-0080D Photodiode Array Spectrophotometer (Hitachi Ltd., Tokyo, Japan). Optical characteristics were recorded using an Edinburgh Instrument F920 spectrometer (Edinburgh Instruments Ltd., Livingston, UK) equipped with 450 W Xenon lamp as excitation source. The monochromator of the F920 spectrometer was incremented over the spectral range to generate continuous monochromatic light for the wavelength-dependent photoresponse assessment. A calibrated power energy meter was used to monitor the optical power output from the monochromator. Devices were tested at RT with DC current-voltage (I-V) measurements using the Keithley semiconductor characterization system model 4200-SCS (Keithley Instruments, Cleveland, OH, USA) coupled with an EP6/Suss MicroTec microprober. A light-emitting diode (LED) solar simulator lamp, class ABA (model LSH 7320, Newport, RI, USA), was used as illumination source. The light intensity of the lamp was uniformly reduced by the neutral density filters. Intensity modulated photocurrent spectroscopy (IMPS) and photovoltage spectroscopy (IMVS) spectra were recorded in the frequency interval from 1 Hz to 1 MHz using an Autolab 302N (Metrohm, Utrecht, The Netherlands) equipped with FRA 32M at two wavelengths of 470 nm (blue) and 627 nm (red) using calibrated LEDs. The light power intensity was fixed at 18 mW with 10% modulation intensity by controlling the applied current in the NOVA software interface.

## 3. Results and Discussion

**Figure 1:**
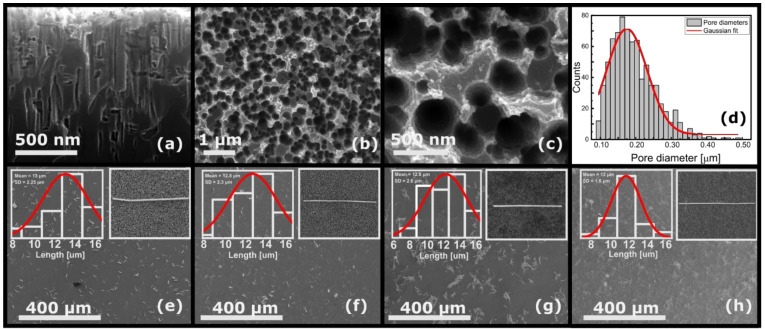
(**a**) Side-view and (**b**) top-view SEM images of the vertical etched Si layer. (**c**) Different-sized Si nanoholes arrayed inside the silicon windows. (**d**) Statistics of Si nanoholes’ morphology, and four different types of NWs deposited on the SiNHs’ surface (inset: SEM image of a single nanowire and nanowires length distribution): (**e**) AgNWs, (**f**) AuNWs, (**g**) NiNWs and (**h**) PtNWs, respectively.

The FESEM images in Figure 1 show the morphology of silicon nanoholes which were further incorporated into SiNHs/MeNWs Schottky photodiode. Figure 1a,b shows the side-view and top-view images of the rough surface of silicon with densely placed nanoholes formed when Ag nanoparticles reacted with the etching solution. The side view reveals the thickness (~1.3 μm) of the nanostructured layer made of parallel cylindrical pores, lined perpendicular to the active surface. Figure 1c indicates the formation of densely distributed nanohole-like cavities with an average diameter of 200 nm as revealed by the histogram of the size distribution presented in Figure 1d.

Further images show the SiNHs’ active area decorated with four types of high aspect-ratio MeNWs. The insets of (Figure 1e–h) correspond to the following metallic nanowires: AgNWs, AuNWs, NiNWs and PtNWs, respectively. In addition, a statistical analysis using ImageJ software [37] was performed to obtain the mean value of the nanowires’ length and the resulting distributions are presented as insets in Figure 1e–h. It was found that the mean value of the length for all nanowires was 12 μm with a standard deviation (SD) of ±2 μm. The mean tip diameter was found to be roughly 207 nm for all nanowires. Further investigation of the SEM micrographs using the binarized image revealed that roughly 6% of the entire SiNHs’ surface represented the total area covered with MeNWs, regardless of the type of metallic nanowire. Given the proportion of nanowires’ coating compared to the photodetector active area, the inherent variations of the contact quality on the full length on the nanowires could be neglected. The deposition parameters, including deposition time and MeNWs concentration, were well controlled and the distribution of MeNWs as single and bundled nanowires could be distinguished on SiNHs’ substrate. It is difficult to completely eliminate the agglomeration of nanowires due to the level of interaction between them, which is influenced by many factors such as the magnetic properties (e.g., Ni) or the specific chemical environment during the fabrication phase. Another factor could be related to the surface tension occurring during solvent evaporation in the solution-based deposition process [38].

**Figure 2:**
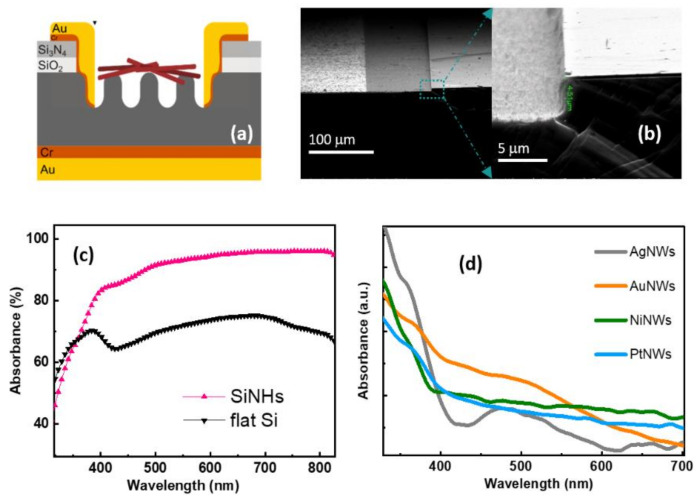
(**a**) Schematic diagram of the optical device based on MeNWs/SiNHs heterojunction. (**b**) Tilt-view SEM image of top Au/Cr layers contacting the SiNHs (left side) and the magnified image of the selected area showing the depth of the oxide window (right side). (**c**) Absorbance spectra of the silicon surface before and after nanoholes’ formation (**d**) and optical absorbance spectra of the colloidal metallic nanowires.

Figure 2a depicts the schematic diagram of a MeNWs/SiNHs-based device and Figure 2b presents the FESEM micrograph of the Au/Cr/SiNHs interface with Au/Cr layers contacting the SiNHs formed inside the squared window at 4.5 μm depth. Diffuse reflectance spectroscopy revealed the wavelength-dependent optical absorbance of the SiNHs and flat Si shown in Figure 2c. The absorbance features of the sensitive surface area of the photodetector revealed not only the operating wavelength regions but also the efficiency of photon absorption. It is well known that silicon is a reliable platform for photonic application due to its highly efficient light absorption over broad Vis–NIR wavelengths. On the other hand, nanostructuring offers the possibility to enhance even more the Si broadband optical absorption. Thus, the Si nanoholes with ~1.3 μm depth yielded an 83–95% flat absorption maximum over the visible spectrum with a more than 20% increase compared to the initial Si surface, indicating the significant reduction of optical reflection achieved. The nanoholes array geometry is an effective way to increase the optical path in Si through trapping and bouncing the light back and forth between the walls of nanoholes [39]. Moreover, the light wavelength when comparable to the nanoholes’ structure makes an important contribution to the light-scattering phenomenon. The SiNHs’ enhanced absorption values and light harvesting ability increases the photocurrent density and ultimately the photodetector performance.

Metallic nanowires’ light absorption performance is also observable in the ultraviolet and visible domain of the spectrum. The collective oscillation of electronic charge density coupled to the electromagnetic field causes a strong resonant enhancement of the local field around the nanoparticle at specific frequencies, known as the localized surface plasmon resonance effect (LSPR) [40]. Ag, Au, Ni and Pt nanostructures with comparable size and shape give rise to similar LSPR features, as shown in the normalized absorbance spectra presented in Figure 2d. All spectra showed a strong LSPR peak positioned around 360–370 nm, which is the primary dipolar transverse mode of surface plasmons ascribed to the short axis of nanowires. The longitudinal plasmon resonance mode is not present due to their high aspect ratio [41]. For AgNWs and AuNWs, a secondary surface plasmon absorption band is observable in the Vis region. The broad and intense SPR band assigned to the anisotropic Au nanowires array covers the whole visible spectrum. Furthermore, the peak corresponding to AgNWs occurs at 480 nm wavelength and may be a result of quadrupole resonance and the interaction with each other, such as electromagnetic coupling between nanowires in close proximity [42].

**Figure 3:**
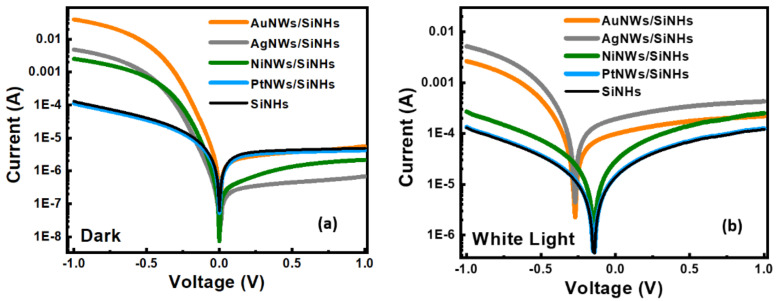
(**a**) The semilogarithmic plots of the forward and reverse bias current-voltage characteristics of the photodetector based on SiNHs and AgNWs/SiNHs, AuNWs/SiNHs, NiNWs/SiNHs and PtNWs/SiNHs heterojunctions under dark condition and (**b**) under white light illumination.

Current-voltage measurements of the fabricated samples were performed at room temperature, both in dark and white light (WL) illumination conditions at 100 mW/cm^2^ intensity, by sweeping the voltage between −1 ÷ 1 V. During the measurements, the voltage was applied on the top contact whereas the bottom contact was connected to be grounded. Consequently, the test samples were biased from the forward (negative voltages) to the reverse (positive voltages) region in order to evaluate both the photocurrent and the electrical parameters of the Schottky diode. The results were plotted on a semilogarithmic scale and are presented in Figure 3a–d.

All tested samples exhibited a typical Schottky diode-like behavior while the MeNWs deposited on the SiNHs substrate distinguished the rectifying performances. Moreover, as can be observed, the investigated devices presented self-powered operating properties demonstrated by the high values of the photocurrent (PC) at zero bias. The reference structure (bare SiNHs) had the photocurrent value of ~ IPC = 1.3 × 10^−5^ and the dark current value of ~ I_D_ = 6.1 × 10^−8^ A, which led to the photo-to-dark current ratio (I_PC_ − I_D_)/I_D_ of ~ 2.1 × 10^2^ in autonomous operation mode. Sensors based on MeNWs show different photoconduction behavior depending on the type of nanowires. By analyzing the four types of heterojunctions, it can be seen that the strongest effect was driven by Ag nanowires, which increased the photocurrent up to 1.9 × 10^−4^ A and reduced the dark current down to 1.7 × 10^−8^ A. Remarkably, the AgNWs/SiNHs Schottky heterojunction yielded a ratio of photo-to-dark current (I_P_-I_D_)/I_D_ ~1.1 × 10^4^ at zero bias, which was significantly higher compared to the 6.9 × 10^2^, 4.5 × 10^2^ and 2.6 × 10^2^ determined for AuNWs/SiNHs, NiNWs/SiNHs and PtNWs/SiNHs heterojunctions, respectively. Similarly, at 0.5 V polarization the photo-to-dark current ratio increased by 1.4 × 10^0^ times for PtNWs/SiNHs up to 4.8 × 10^1^ times for AgNWs/SiNHs compared to the reference performance.

**Figure 4:**
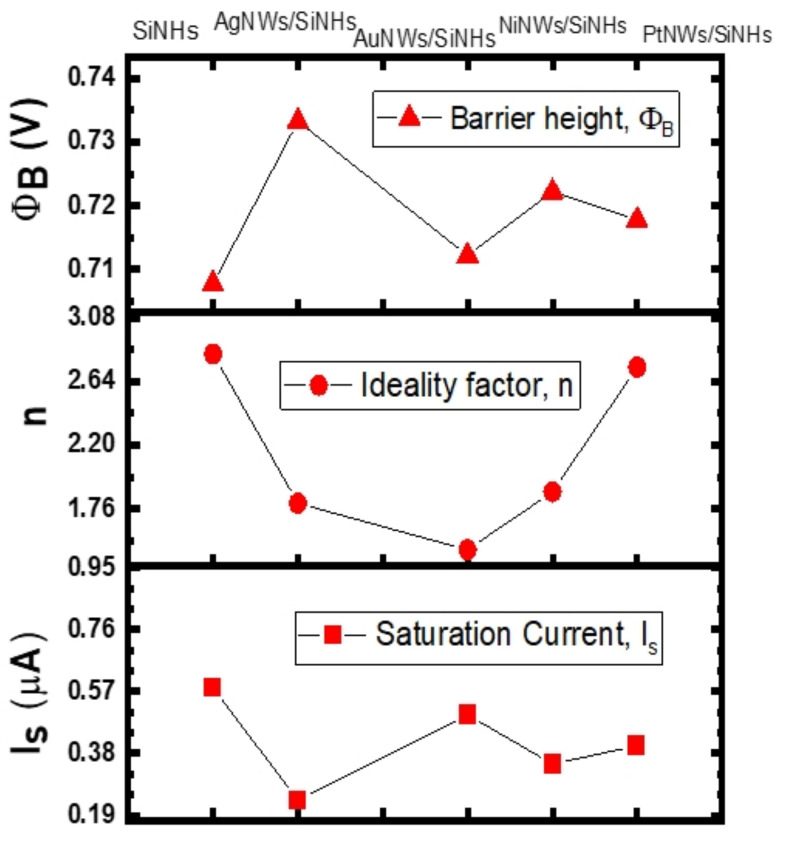
Schottky-diode parameters of MeNWs/SiNHs-based devices.

The dark I-V characteristics of the forward bias region were further used to evaluate the main electrical parameters, such as Schottky barrier height (SBH–Φ_B_), ideality factor (*n*) and saturation current (Is). Based on the thermionic emission law [43], the abovementioned electrical parameters calculated from forward current-voltage characteristics are plotted in Figure 4. The SBH shows close values ranging from 0.709 (SiNHs) to 0.732 V (AgNWs/SiNHs). This fact is strongly supported by the dark I-V characteristics (Figure 3) which have a similar trend at low-voltage bias domain. The deviation from the ideality is given by *n*, a factor that offers information about the rectifying performances of the Schottky diode. In this case, *n* starts from a value of 2.83 for the SiNHs reference and decreases towards the unity after the deposition of the MeNWs, reaching its lowest value *n* ~1.48 in the case of the AuNWs/SiNHs sample. This is in strong correlation with the I-V characteristics, where the AuNWs/SiNHs sample shows a significant variation of the forward current. The improvements of electrical parameters in dark conditions can be attributed to surface passivation of the SiNHs leading to an electrically homogenous active area. By directly contacting the Si, MeNWs might operate as trapping centers for the minority carriers, thus minimizing recombination loss. The saturation current was also determined in order to estimate the dark current for the fabricated samples and is related to the number of charges that overcome the barrier under reverse bias. As one can see in Figure 4, the saturation current varied between 0.581 µA (reference sample) up to 0.237 µA (AgNWs/SiNHs sample), showing a decrease by more than half in the dark current when the AgNWs were used. This result is also in strong correlation with the reverse I-V characteristics presented in Figure 3, where we can easily observe a dark current suppression for the AgNWs/SiNHs sample.

**Figure 5:**
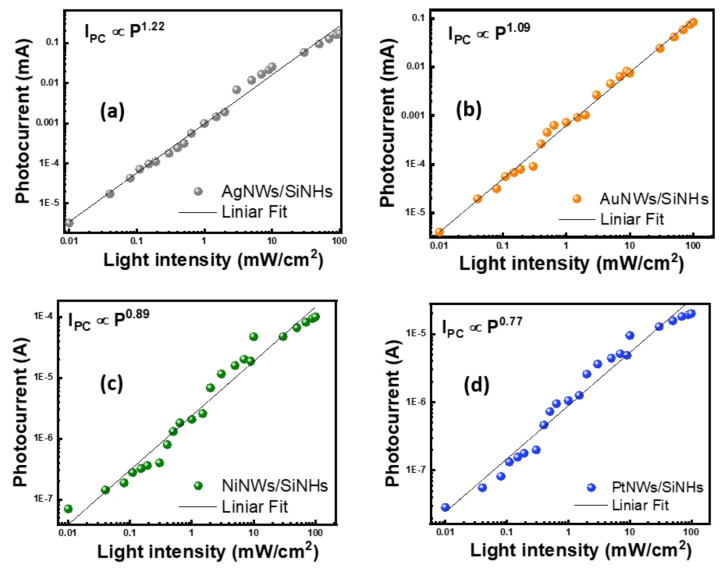
(**a**) Photocurrent dependence on illumination intensity (white light) measured at zero applied voltage of photodetectors based on AgNWs/SiNHs, (**b**) AuNWs/SiNHs, (**c**) NiNWs/SiNHs and (**d**) PtNWs/SiNHs heterojunctions.

Next, the photoresponse of the devices based on MeNWs/SiNHs was measured under zero bias conditions by applying different optical powers varying from 0.01 to 100 mW cm ^−2^ (Figure 5a–d). The photocurrent response showed a strong intensity-dependent behavior with a good linearity from lowest to highest power region. The dependence was fitted by a power law (I_PC_ ∝ P^θ^), where P is the light power and θ represents its exponent correlated to the photoresponse, with relatively large exponent values varying from 0.77 for PtNWs/SiNHs to 1.22 for AgNWs/SiNHs, whereas the greater than 1 value for the exponent is associated with dynamic plasmonic effects. In the framework of Rose theory [44], such close to integer values reveal the photoresponse is mostly proportional to the photogenerated carriers with a very low limitation caused by defect states or trapping centers. The results indicate MeNWs/SiNHs-based devices are able to operate in self-bias mode with a broad linear characteristic of the photoresponse, which is suitable for a variety of visible photodetection applications.

The linear dynamic range (LDR) is a critical figure of merit parameter of the photodetector, which is based on the relation between the highest and lowest photocurrent values situated in the linear photoresponse region, according to the fallowing equation:


(1) $$\mathrm{LDR}=20\log[I_{\text{max,liniar}}/I_{\text{min,liniar}}]$$

The AgNWs/SiNHs device possessed the highest LDR value of 94 dB within 0.01 mW cm^−2^ and 100 mW cm^−2^ illumination power domain, while in case of AuNWs/SiNHs the LDR values were slightly decreased to 86 dB for the same conditions.

**Figure 6:**
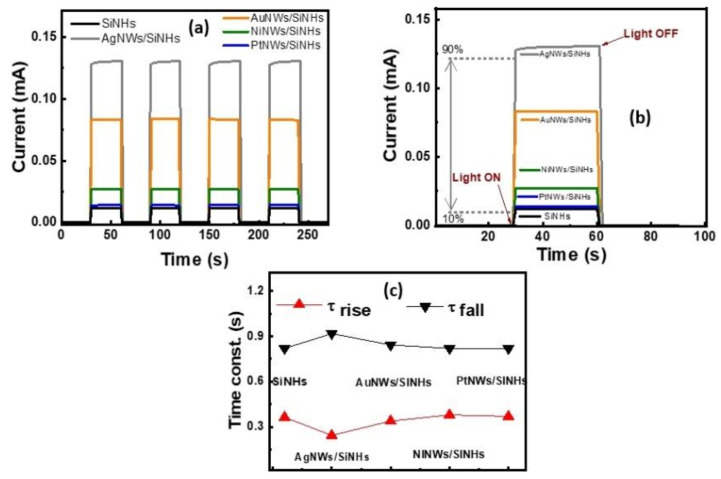
(**a**) Self-biased photocurrent response of the MeNWs/SiNHs photodetectors under on/off WL illumination. (**b**) Response time plots used to calculate the time constants. (**c**) The rise and fall time constants corresponding to each type of heterojunction.

Furthermore, transient photocurrents (Figure 6a) were measured at zero bias under 100 mW cm^−2^ illumination, alternatively turned on and off in order to demonstrate the stability of the photocurrent responses. The photocurrent response of the bare and MeNWs-modified SiNHs photodetectors was repeated for many on/off cycles and exhibited a retention of more than 90% of the initial WL illumination photoresponse after 100 cycles, which demonstrates a good operation stability. The stability of the transient photoresponse on/off characteristics of the reference device was not altered by the surface modification of SiNHs with metallic nanowires. Two time constants were estimated, the rise time (τ_r_), which is the time taken for the photocurrent to rise from 10% to 90% of the saturation value, and the fall time (τ_f_), which is the time taken for the photocurrent to fall from 10% to 90% of the saturation value, as plotted in Figure 6b. The AgNWs/SiNHs device showed the fastest rise time of τ_r_ ~ 244 ms and the longest fall time of τ_f_ ~ 918 ms, whereas for the AuNWs/SiNHs device the rise time and the fall time were τ_r_ ~339 ms and τ_f_ of ~ 843 ms, respectively (Figure 6c). The time constants are strongly influenced by the lifetimes of plasmon-induced charge carriers injected at the Me/Si interface. On the other hand, NiNWs and PtNWs-based devices demonstrated very small variations of rise time and fall time compared to the bare SiNHs reference.

**Figure 7:**
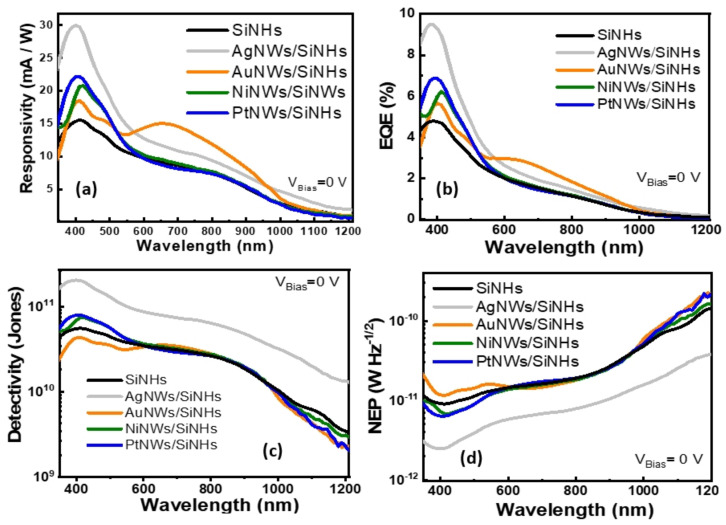
(**a**) Wavelength-dependent responsivity spectra; (**b**) external quantum efficiency spectra; (**c**) specific detectivity spectra and (**d**) noise-equivalent power (NEP) of MeNWs/SiNHs-based photodetectors under self-biased conditions.

Figure 7a–d shows responsivity (R), external quantum efficiency (EQE), detectivity (D), and the noise equivalent power (NEP) plotted as a function of illumination wavelength. They represent important figure of merit parameters to evaluate and compare the performance of the self-biased MeNWs/SiNHs-based photodetectors.

The responsivity spectra R is the ratio of the measured photogenerated current (I_PC_) to the incident illumination power (P_i_) on the optical active area (A) and gives the light/dark gain of the detector:

(2) $$R=\frac{I_{PC}}{P_{i}\times A}$$

Remarkably, the responsivity (Figure 7a) of the AgNWs/SiNHs device showed a peak value of ~30 mA/W at 400 nm exhibiting 2-fold enhancement while the AuNWs/SiNHs device responsivity exhibited a maximum 1.6-fold increase at 650 nm, reaching 15 mA/W for the same illumination conditions and compared with the reference. The responsivity values reported in the present study are larger than previously reported for metallic nanowire photodetectors [45,46,47,48].

The metallic nanowires’ discrete distribution allows the incident light to be absorbed by the Si surface, where charge carriers are mostly photogenerated then separated as e-h pairs and collected at Au/Cr electrodes. In this system, each type of Me nanowire participates not just as a heterojunction component with excellent electrical conductivity enabling the carrier injection/extraction process, but also demonstrates a key role in enhancing the selectivity over a broadband domain. The enhancement of photoresponse observed over specific wavelengths is a direct indicator of the increased photogenerated carriers associated with a specific plasmonic absorption domain depending on nanowires’ composition, size or morphology. A more intense LSPR effect produces a larger enhancement of the overall photocurrent derived from a combination of plasmonic effects following light and MeNWs’ interactions, such as non-radiative hot electrons generation and injection into the Si surface combined with induced local electromagnetic field enhancement surrounding the metal nanowires, and plasmonic heat effects [14]. The responsivity spectra confirm the potential of MeNWs/SiNHs to work as photodetectors for tunable sensitivity applications.

The external quantum efficiency (EQE) figure of merit (Figure 7b) expresses the collection efficiency of photogenerated charges created at specific wavelengths. Calculations were performed according to the fallowing equation,

(3) $$\mathrm{EQE}=\frac{hc}{e}\;\frac{R}{\lambda }$$

where h is the Planck constant, c is the speed of light, e is the electronic charge and λ is the incident light wavelength. The highest EQE value of the self-driven devices reached 9.3% in 400 nm for AgNWs/SiNHs while another observable peak of 2.8% was situated at about 650 nm for AuNWs/SiNHs devices. The EQE increased up to 97% in 400 nm due to AgNWs and attained better performance compared to the reference for all the illumination wavelengths.

Figure 7c,d shows the detectivity and NEP as a function of incident wavelength as well. The parameters were calculated with the fallowing relations:

(4) $$\begin{array}{l} D^{*}=\frac{R}{\sqrt{\frac{2eI_{dark}}{A}}}\\ \mathrm{NEP}=\frac{\sqrt{S}}{D^{*}} \end{array}$$

NEP is used to evaluate the weakest detectable signal, competing with the noise, that can be revealed, and detectivity (D^*^) is related to device sensitivity and to the inverse of the smallest detectible signal. The best detectivity and NEP values were obtained for the AgNWs/SiNHs device, which had the highest D value of ~2 × 10^11^ Jones and, subsequently, the lowest NEP value ~2.4 × 10^−12^ WHz^−1/2^ obtained at 400 nm, which is the wavelength of the maximum responsivity peak. In our case, the AgNWs/SiNHs was the most sensitive device. 

To clarify the features obtained in the responsivity spectra, the wavelength-dependent photocurrent enhancement, and for in-depth understanding of the associated opto-electrical phenomena, non-steady state intensity modulated photocurrent spectroscopy (IMPS) and intensity modulated photovoltage spectroscopy (IMVS) techniques were further employed at two incident light wavelengths [49]. The most prominent responsivity values obtained for the MeNWs/SiNHs devices were situated around ~400 and ~650 nm. Therefore, the IMPS and IMVS spectroscopy measurements were performed under 470 and 627 nm illumination wavelengths corresponding to the available LEDs wavelengths.

**Figure 8:**
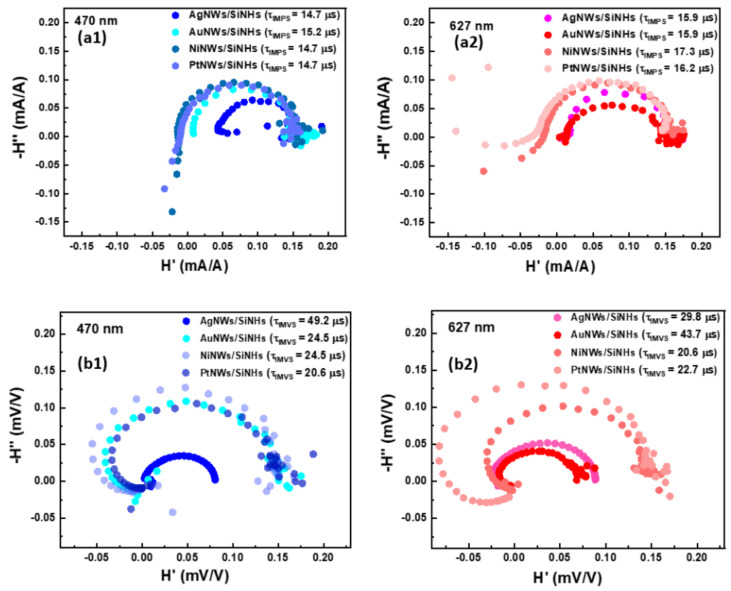
(**a1**,**b1**) IMPS and IMVS responses in the complex plane at 470 nm and (**a2**,**b2**) at 627 nm respectively.

Analyzing together IMPS and IMVS data, we can establish an image of the processes which take place in devices, from the excitation of the electron-hole pair to the charge separation and transport, including recombination and trapping/de-trapping processes during the charge transit until their extraction [50]. The IMPS measurements on the other hand provide practically an image of the charge carrier dynamics and recombination kinetics in the interfaces; the IMVS, carried out in open-circuit conditions when no charge is extracted, is associated with recombination processes. Figure 8 presents typical IMPS and IMVS plots obtained at the same light intensity at two wavelengths of interest, namely 470 and 627 nm. The existence of only one arc presented in the IMPS spectrum indicates that the photoexcited carriers accumulated at the device surface are not lost in the interface recombination centers and are transferred to participate in the charge transport. 

The charge transfer time can be estimated from the equation $\tau_{tr}=\frac{1}{2\pi f_{IMPS}},$ where f_IMPS_ is the characteristic frequency at the minimum of the IMPS imaginary component [51]. As expected, the resulting times were almost similar for all devices when they were illuminated with modulated light, with values estimated between 14.7–15.2 µs at blue illumination and 15.9–17.3 µs at red light, respectively, indicating that the change in the charge-transfer kinetics was negligible regardless of the nature of plasmonic nanowires used to modify the surface. Moving on to the IMVS spectra recorded for MeNWs/SiNHs devices, a more substantial difference between the individual plots was observed, with an increased range of variation of the characteristic recombination times estimated from the equation $\tau_{rec}=\frac{1}{2\pi f_{IMVS}}$, where f_IMVS_ is the characteristic frequency at the minimum of the IMVS imaginary component [52]. Significantly higher times were calculated from IMVS measurements, especially for AuNWs/SiNHs and AgNWs/SiNHs devices, indicating slow charge recombination processes. It is interesting that roles changed between these two devices depending on the illumination wavelength. Therefore, if under the blue light AgNWs/SiNHs devices had two times longer carrier lifetime compared with the rest of devices, while under the red light AuNWs/SiNHs showed the slowest recombination process and a diminished loss of carriers, in accordance with optical responsivity measurements; yet, it has to be noted that the difference between the two lifetimes was not so high as in the previous case.

The significant enhancement of the photoresponse was further validated by COMSOL simulation, which provided valuable insight into the electromagnetic field enhancement phenomenon taking place at the SiNHs surface. Thus, finite element method simulations were performed in commercial software COMSOL Multiphysics.

**Figure 9:**
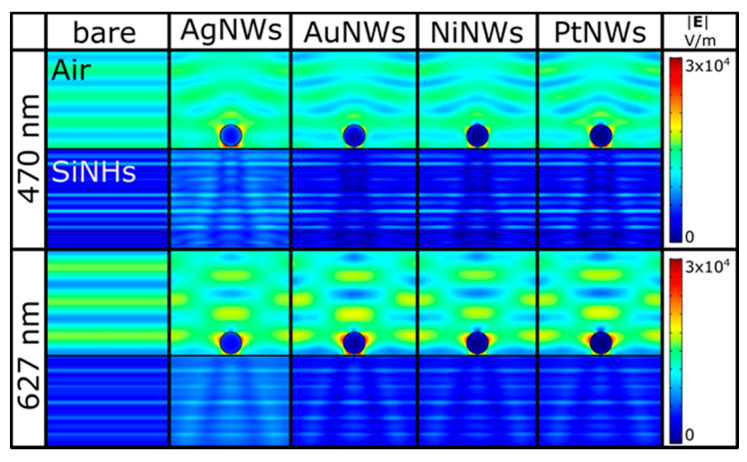
Electric field distributions around metallic nanowires on the SiNHs surface owing to the localized surface plasmon resonance (LSPR) response obtained at 470 nm and 627 nm illumination. For comparison, the bare SiNHs surface is also displayed in the first column.

First of all, the optical properties of SiNHs were found by employing the Bruggeman effective medium theory [53] with a fill ratio of 40% obtained from the SEM micrographs. Secondly, the refractive indices of the utilized nanowires (Au, Ag, Ni and Pt) and silicon were taken from literature [54,55]. The simulations were performed at the same wavelengths as IMPS and IMVS measurements: 470 and 627 nm. The incident light was linearly polarized along the nanowire axis and came from a domain with a refractive index of 1 (air). Periodic boundary conditions were used to take into account the neighboring nanowires, and perfectly matched layers, to avoid any unwanted reflections from the substrate backside. A comparison of the SiNHs surface before and after metallic nanowires addition for 470 nm and 627 nm incident wavelength can be seen in Figure 9. A plasmonic Schottky optical detector relies on plasmonic nanostructures for local electric field enhancement as the main mechanism responsible for the generation of hot carriers that contribute to the photogenerated current and, subsequently, to responsivity behavior.

It was found that the device fabricated with Ag nanowires had the highest value of responsivity, reaching ~30 mA/W, whereafter the responsivity followed a descending trend for Au, and Ni and Pt nanowire devices. The same trend was demonstrated by the electric field distribution simulation at 470 nm presented in Figure 9 (first row) where the highest local field enhancement was obtained for the silver nanowire configuration with a value of 3.58 × 10^4^ V/m. Additionally, the plasmonic effects successfully increased the responsivity of SiNHs photodetectors in the 550–960 nm domain in the gold nanowires configuration, as shown by the increased electric field distribution around the Au nanowires (Figure 9 second row). The highest electric field at 627 nm was obtained for the Au nanowires with a value of 2.91 × 10^4^ V/m, succeeded by silver nanowires with 2.73 × 10^4^ V/m and afterwards by the NiNWs and PtNWs, which confirms the reduced plasmonic effect of the last two types of nanowires on SiNHs device performance.

## 4. Conclusions

In summary, a novel self-powered Schottky photodetector architecture based on silicon nanoholes and four different metallic nanowires incorporated on the Si surface were extensively investigated. A plasmon-enhanced broadband photoresponse over UV, visible and near infrared optical domains was achieved mainly for Ag and Au nanowires, which demonstrated much stronger local effects than their Ni and Pt nanowires counterparts. Notably, the self-biased AgNWs/SiNHs device increased the photo-to-dark current ratio by two orders of magnitude and showed the highest responsivity of ~30 mA/W and detectivity of ~2 × 10^11^ Jones and the lowest NEP of ~2.4 × 10^−12^ WHz^−1/2^ at 400 nm, whereas the AuNWs/SiNHs device had a significantly enhanced photoresponse over a very broad wavelength domain between 550 and 960 nm. Moreover, the dynamic of electron recombination processes revealed by IMVS measurements confirmed the wavelength-dependent photoresponse of AgNWs and AuNWs devices. The increase in sensitivity and photoresponse was explained by plasmonic absorption, hot electron injection and local field enhancement effects at the MeNWs and SiNHs interface as confirmed by numerical simulation. This work demonstrates that integrating engineered plasmonic interfaces based on metallic nanowires is a promising route to increase the performance of photodetection, sensing and solar cell applications.

## Data Availability

Data is contained within the article.
